# Regulation of the Anti-Inflammatory Cytokines Interleukin-4 and Interleukin-10 during Pregnancy

**DOI:** 10.3389/fimmu.2014.00253

**Published:** 2014-05-27

**Authors:** Piyali Chatterjee, Valorie L. Chiasson, Kelsey R. Bounds, Brett M. Mitchell

**Affiliations:** ^1^Department of Internal Medicine, Texas A&M Health Science Center, Temple, TX, USA; ^2^Baylor Scott and White Health, Temple, TX, USA

**Keywords:** inflammation, pro-inflammatory cytokines, anti-inflammatory cytokines, pregnancy disorders, immune cells

## Abstract

Inflammation mediated by both innate and adaptive immune cells is necessary for several important processes during pregnancy. Pro-inflammatory immune cell activation plays a critical role in embryo implantation, placentation, and parturition; however dysregulation of these cells can lead to detrimental pregnancy outcomes including spontaneous abortion, fetal growth restriction, maternal pathology including hypertensive disorders, or fetal and maternal death. The resolution of inflammation plays an important role throughout pregnancy and is largely mediated by immune cells that produce interleukin (IL)-4 and IL-10. The temporal and spatial aspects of reducing inflammation during pregnancy represent a complex process that if not functioning optimally can lead to persistent inflammation and pregnancy complications. In this review, we examine how immune cells that produce IL-4 and IL-10 are regulated throughout pregnancy as well as the effects that reduced IL-4 and IL-10 signaling has on fetal and maternal physiology.

## Introduction

The immunological features of normal pregnancy are unique as the maternal immune system has to accept a semi-allogeneic fetus, a product of two histo-incompatible individuals. Medawar proposed that in order to accept a half-foreign fetus, the mother needs to be in an immunosuppressed state (1). Recent progress in our understanding suggests that the maternal immune system not only needs to be suppressed but at the same time also needs to protect the mother and the growing fetus from infection during pregnancy. Thus, a successful pregnancy depends on the ability of the mother’s immune system to become tolerant to paternal antigens as well as the ability to reject the fetus in case of pathogen infection.

The maternal immune response is regulated by a complex array of cytokines to protect the conceptus and promote proper growth and development of the placenta. Wegmann and colleagues suggested that during pregnancy there is a T-helper (Th) 2 bias to promote tolerance to the half-foreign fetus and Th1 cytokines are detrimental to the tolerance of the conceptus, similar to allografts in transplant recipients (2–5). It has been found that during tolerance induction to an allograft there is a decrease in Th1 cytokines such as interleukin (IL)-2 and IFNγ and an increase in Th2 cytokines including IL-4 and IL-10. Conversely, high levels of IL-2 and IFNγ were detected in rejecting allografts (6–8). Existing data suggest that Th2 bias in pregnancy is an oversimplified model and that during the various stages of pregnancy the pro-inflammatory and anti-inflammatory cytokine milieu is dynamically modulated. The first stage of pregnancy, which involves a blastocyst implanting into the uterus, is a predominantly pro-inflammatory phase. Localized activation of inflammatory mediators occurs and the mother’s immune system repairs the damage done by the invading blastocyst. The second phase of pregnancy is a predominantly anti-inflammatory phase. Th2 cytokine skewing during the second phase of pregnancy can be systemic or local at the feto-maternal interface. The last phase of pregnancy is parturition, which causes contraction of the uterus and again, the pro-inflammatory milieu is predominant. Inflammation is tightly controlled during all stages of pregnancy, however excessive and persistent maternal inflammatory responses are associated with adverse pregnancy outcomes.

Pregnancy disorders such as preterm birth (PTB), fetal growth restriction (FGR), and preeclampsia (PE) are often associated with infection during pregnancy (9–11). Infection due to bacteria, viruses, and parasites, which normally induce a Th1 immune response can impact placental development and function and ultimately fetal survival (12). Th1 responses induce IFNγ that in turn propagates Th1 responses by up-regulating IL-12 receptor expression and inhibiting Th2 responses.

Preterm birth is associated with increased production of pro-inflammatory cytokines and chemokines such as IL-1β, IL-6, TNFα, and CXCL8 (13). These cytokines induce prostaglandin synthesis in the placental tissues that triggers preterm labor (14, 15). Likewise, maternal inflammation due to infection is an important contributor to the development of FGR (16). Administration of the anti-inflammatory cytokine IL-10 can attenuate FGR induced in rats by lipopolysaccharide (LPS) or infection with *E. coli* (17). The addition of exogenous IL-10 reduces fetal resorption in pregnancies of CBA/J × DBA/2 mice. Anti-IL-10 neutralizing antibodies increase fetal loss and lead to growth defects after birth (18, 19). PE is also associated with an exaggerated maternal inflammatory response. Zenclussen and colleagues demonstrated that adoptive transfer of the Th1 cells into pregnant mice was associated with the development of PE-like symptoms (20). Pro-inflammatory cytokines are not only increased in PE but the production of the anti-inflammatory cytokines IL-4 and IL-10 are also known to be decreased. In this review, we highlight how immune cells that produce IL-4 and IL-10 are modulated during pregnancy and their role in adverse pregnancy outcomes.

## Immune Cells That Produce IL-4 and IL-10 and Their Actions

Interleukin-4 and IL-10 are pleiotropic anti-inflammatory cytokines that function mainly by suppressing the pro-inflammatory milieu. Several different immune cells that produce IL-4 are activated T cells, mast cells, basophils, eosinophils, and NKT cells (21, 22). IL-4 aids in the polarization of antigen-stimulated naïve Th cells into Th2 effector cells as well as propagates Th2 responses by binding to its receptor, IL-4Rα, and activating the signal transducer and activator of transcription (STAT) six signaling pathway (23–26). STAT6, through the induction of a zinc-finger transcription factor GATA3 (GATA-binding protein 3), might directly suppress Th1 cell development by silencing IFNγ expression (27). Recent studies also indicate that IL-4 enhances Th2 immunity by inhibiting Th1 responses through the repression of IL-12 signaling (28). Several studies implicate a role for IL-4 in regulatory T cell (Treg) development and maintenance IL-4 signaling through STAT6 is important for FoxP3 mRNA expression and protein production in natural Tregs (29–32). IL-4 also induces the formation of inducible Tregs from naïve CD4+ T cells. Thus, IL-4 not only mediates Th2 cell function but also plays a part in the regulation of Tregs which play an important role in successful pregnancies.

Interleukin-10 production was first determined in Th2 cells and was initially thought to be only produced by immune cells, but later studies demonstrated that IL-10 is also produced by non-immune cells (33). Immune cells that produce IL-10 include subsets of T cells such as Th1, Th2, and Th17, as well as monocytes, macrophages, dendritic cells, human B cells, granulocytes, eosinophils, and mast cells. Non-immune cells that produce IL-10 include keratinocytes, epithelial cells, and tumor cells. IL-10 primarily exerts its anti-inflammatory effect by inhibiting pro-inflammatory cytokines such as IL-1, IL-6, IL-12, and TNF as well as chemokines (34). IL-10 also inhibits antigen presentation by blocking MHC class II expression and co-stimulatory molecules such as CD80 and CD86 (35). IL-10 exerts its biological effect by binding to its receptor which is composed of two subunits, IL-10R1 and IL-10R2 (35). Initially, IL-10 binds its cognate receptor IL-10R1 and the binding of IL-10R2 is specific to initiate a signaling cascade. IL-10 then activates Janus kinase (JAK) and STAT pathways. This recruits Tyk2 and Jak1 to the receptor complex and induces phosphorylation of the receptors leading to transcription of IL-10-regulated genes (36). IL-10 production is also associated with other types of immune cells such as macrophages and myeloid-derived suppressor cells (MDSCs). Following Toll-like receptor (TLR) activation in macrophages and MDSCs, the signaling cascade comprising the adaptor molecule TIR-domain-containing adaptor protein inducing IFNβ (TRIF) is activated. The extracellular signal-regulated kinase 1 (ERK1), ERK2, p38, and nuclear factor-κB (NF-κB) pathways are activated leading to the production of IL-10 and several other genes. IL-10 up-regulates its own production by modulating tumor progression locus 2 (TPL2) expression (34). Both IL-4 and IL-10 mediate signaling between immune cells and also regulate recruitment, activation, and suppression of both immune and non-immune cells.

## Modulation of IL-4 and IL-10 during Pregnancy

Anti-inflammatory cytokines perform a multitude of functions during normal pregnancy by promoting placental formation, modulating trophoblast invasion and differentiation, inducing placental proliferation and angiogenesis, and inhibiting pro-inflammatory cytokines. IL-4 is detectable at the feto–maternal interface during all phases of pregnancy (37). IL-4 is produced not only by immune cells of the placenta but also by the maternal decidua, amniochorionic membranes, cytotrophoblasts, and both maternal and fetal endothelial cells (38, 39). IL-4 production is increased in the gravid state and levels of IL-4 increase throughout normal pregnancy (40). Progesterone is a known inducer of IL-4 and together they act to inhibit Th1 responses during pregnancy. Given the important role of IL-4 in suppressing inflammation, it is surprising that IL-4-knockout mice have normal pregnancies with respect to fetal growth and development (41). This would suggest that the role of an individual cytokine may not be crucial to the success of pregnancy but rather depends on the complex interplay with other cytokines in a spatiotemporal manner.

Interleukin-10 has been shown to be constitutively expressed in placental villous trophoblasts but not in extravillous trophoblasts (42). Additionally, uterine NK cells (uNK cells), monocytes, and Tregs in the decidua are also important producers of IL-10 (36). IL-10 acts on its receptors (IL-10R) that are expressed on several cell types including placental trophoblasts, decidual stromal cells, macrophages, and uNK cells. In mice, IL-10 is expressed throughout pregnancy and peaks at gestational day 12 (37). To determine the exact role of IL-10 in pregnancy, pregnant IL-10^−/−^ mice were compared to pregnant wild type (WT) mice and no change in litter size or development were noted indicating that IL-10 is not essential for pregnancy (41). However, IL-10 plays a role in placental growth and remodeling because IL-10^−/−^ mice exhibited increases in placental size and maternal blood sinuses (43). IL-10 is not essential for the growth and development of the fetus in mice but rather it plays an important role to inhibit excessive inflammation. Pregnant IL-10^−/−^ mice are susceptible to low doses of LPS and CpG (a TLR 9 agonist) compared to WT mice (44, 45). These results suggest that IL-10 acts as a protective agent during infection and deficiency of IL-10 exacerbates inflammation in mice. Normal pregnant women were determined to have increased IL-10 production during the first and second trimesters but not in the third trimester (46). Moreover, IL-10 production decreases prior to labor and delivery of the fetus and placenta and increases post labor (47). Precise regulation of IL-4 and IL-10 are important to curtail maternal inflammation and allow crosstalk between the placental decidua and the invading fetal trophoblasts at different stages of pregnancy.

## IL-4 and IL-10 in Spontaneous Abortion and Fetal Growth Restriction

**Figure 1:**
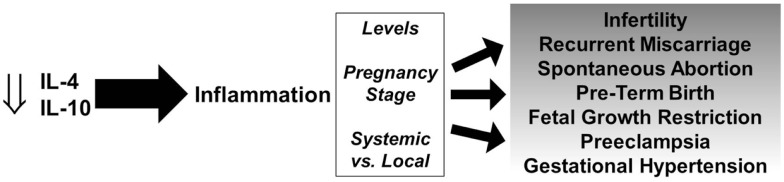
**Role of IL-4 and IL-10 in pregnancy disorders**. Decreased levels of IL-4 and IL-10 promote persistent inflammation and depending on the levels, stage of pregnancy, and systemic vs. local effects, can lead to a spectrum of gestational complications.

It has been well documented that the lack of fetal tolerance is largely mediated by Th1 cells. Their recruitment from the maternal circulation into the feto–maternal interface and their production of pro-inflammatory cytokines coupled with a decrease or lack of increase in anti-inflammatory cytokines can lead to a spectrum of pregnancy disorders (Figure 1). Various cells that produce IL-4 and IL-10 including NK cells, T cells, regulatory B cells, and others are dysregulated and fail to increase production of these anti-inflammatory cytokines at the appropriate time and location.

Immunological models of spontaneous abortion and FGR in animals include the female CBA/J × male DBA/2J mating with and without stress, excessive TLR activation in early pregnancy (LPS and poly I:C), and transvaginal rIL-17 administration, and all of these are associated with decreased production of IL-4 and IL-10. Several studies have reported that abortion-prone CBA/J females demonstrate decreased circulating and placental levels of IL-4 and IL-10 (18, 48–52) and that experimental therapeutics at various time points including a B7 monoclonal antibody, adenoviral-mediated heme oxygenase-1 overexpression, progesterone or its derivatives, adoptive transfer of Tregs, or alloimmunization, all decrease abortion rate and this is associated with increased IL-4 and IL-10 and/or Th2/Th1 ratios (18, 48–50, 52–55). A more direct study demonstrated that administration of IL-4 as well as IL-4 and IL-10 decreased the resorption rate in these mice (51). In animals, TLR activation in early pregnancy induces spontaneous abortion or resorption and this is regulated by IL-10 as rIL-10 administration was able to prevent these effects (18, 56–59). Further support for IL-10 and its role in preventing spontaneous abortion was demonstrated in mice administered rIL-17 transvaginally on gestational day 1 which decreased decidual IL-10 levels and induced abortion (53). Adoptive transfer of IL-10-producing Tregs from pregnant mice increased IL-10 levels and decreased abortion rates in these mice (53).

These experimental studies are supported by clinical observations in women who have had recurrent or initial reproductive failures. Production of IL-4 and IL-10 by NK cells, regulatory B cells, T cells, and others is decreased in women who have had spontaneous abortions, recurrent miscarriages, small for gestational age babies, and infertility. Several studies have reported low levels of IL-4, IL-4-producing cells, and Th1 cytokine/IL-4 ratios in women with spontaneous abortions (60–65). With respect to IL-10, numerous studies have reported low levels of IL-10, IL-10-producing cells, and Th1 cytokine/IL-10 ratios in women with spontaneous abortions (40, 61–71). Additionally, women experiencing multiple unsuccessful *in vitro* fertilization cycles have increased TNFα+/IL-4+ and TNFα+/IL-10+ T cell ratios (65). A recent study found that low levels of circulating anti-inflammatory cytokines during early gestation were associated with habitual miscarriages in women (72). Further support for an important role of IL-4 and IL-10 in preventing reproductive failure was provided by a study in which i.v. Ig therapy in women with recurrent spontaneous abortions increased IL-4 and IL-10 levels and decreased the ratio of IFNγ+/IL-4+ T cells (73). Together these data support the notion that a lack of early, appropriate anti-inflammatory responses and excessive inflammation can lead to reproductive failure. Further experimental and clinical studies in which augmentation of IL-4- and IL-10-producing immune cells would determine whether this would be sufficient to induce successful pregnancies.

## IL-4 and IL-10 in Preeclampsia

One of the characteristics and potential cause in some cases of PE is uncontrolled amplification of the maternal immune system. The excessive pro-inflammatory state seen in PE may be partly due to increased production of pro-inflammatory cytokines and/or decreased production of anti-inflammatory cytokines. Indeed, PE-like symptoms develop in rats following infusion of IL-6 or TNFα indicating that pro-inflammatory cytokines contribute to the development of PE (74, 75). Recent experiments from our lab indicate a pathogenetic role of decreases in the anti-inflammatory cytokine IL-4 in PE. We demonstrated that although fetal growth and development was not affected in IL-4^−/−^ mice, mild PE-like symptoms such as hypertension and proteinuria developed during pregnancy. Additionally, deficiency of IL-4 induced systemic and placental inflammation in mice. These experiments implicate that deficiency of IL-4 contributes to mild cardiovascular and renal effects and the protective role of IL-4 is more pronounced during infection. In support, we demonstrated that pregnant IL-4^−/−^ mice exhibited even further increases in inflammation and PE-like symptoms following viral mimetic activation of TLR3. Importantly, non-pregnant IL-4^−/−^ mice did not exhibit hypertension and proteinuria at baseline or following TLR3 activation (76). These observations also correlate well with numerous clinical studies in which women with PE have been reported to have decreased IL-4 levels and increased circulating levels of the soluble IL-4 receptor compared with normotensive pregnant women (77–79). These studies establish a role for decreased IL-4 in the development of PE and also indicate that administration of IL-4 may be a viable treatment option for women with PE.

Similar to IL-4, several studies document the importance of IL-10 in preventing PE. Serum from PE patients induces the clinical features of PE such as hypertension, proteinuria, and FGR in IL-10^−/−^ mice. PE serum induced HIF-1α in the placenta which may have triggered production of the anti-angiogenic factors sFlt-1 and sEng and also induced renal pathology and poor spiral artery remodeling. Endovascular capillary tube formation is also significantly disrupted by serum from PE patients in these IL-10^−/−^ mice. However, serum from healthy women or women with PE administered to non-pregnant animals failed to induce any PE-like features (80). In another study by Lai et al. deficiency of IL-10 coupled with hypoxia induced severe PE-like features including renal pathology, proteinuria, and hypertension. Moreover, increased expression of anti-angiogenic factors, apoptotic pathways, and placental injury were noted. Expectedly, recombinant IL-10 administration reversed the hypoxia-induced features in pregnant IL-10^−/−^ mice confirming the protective role of IL-10 in PE (81). In our studies, pregnant IL-10^−/−^ mice exhibit mild hypertension, endothelial dysfunction, and proteinuria only during pregnancy. In addition, PE-like symptoms were augmented in IL-10^−/−^ mice following activation of TLR3 during pregnancy (82). Clinical studies further support reduced production of IL-10 from patients with PE (83).

Based on the aforementioned studies, we hypothesized that administration of either of the anti-inflammatory cytokines IL-4 or IL-10 or co-treatment with both recombinant IL-4 and IL-10 may improve outcomes in TLR-activated PE mice. Administration of IL-4, IL-10 alone, or IL-4/IL-10 co-treatment during gestation normalized blood pressure and endothelial function in mice treated with a TLR3 agonist. IL-4/IL-10 co-treatment had the most beneficial effect on fetal development and renal function as well as decreased the levels of the pro-inflammatory cytokines IL-6, IFNγ, and TNFα (84). These studies raise the possibility of using anti-inflammatory cytokines in combination as a therapeutic option for women with PE.

## Conclusion

The role of inflammation is important and necessary for successful pregnancies, however aberrant and persistent inflammation and the lack of resolution by anti-inflammatory cytokine-producing cells can lead to a variety of pregnancy disorders depending on various factors (Figure 1). IL-4 and IL-10 play crucial roles in the success of pregnancy and there is strong evidence that a deficiency in IL-4 and/or IL-10 contributes to infertility, spontaneous abortion, PTB, FGR, and hypertensive disorders of pregnancy.

Most studies to date have aimed to determine the role of each individual cytokine which has generated important findings and improved our understanding of the role of anti-inflammatory mediators during pregnancy; however there is considerable redundancy among cytokines and within an immune response. Integrative studies that take into context the local environment and cytokine milieu, especially during the gravid state, are necessary to determine how cytokine–cell and cell–cell communication influences local and systemic inflammation and the physiological effects during pregnancy. Novel therapies that target the augmentation of multiple anti-inflammatory cytokines including both IL-4 and IL-10 may elicit better effects than a single anti-inflammatory cytokine targeting therapy. The challenge will be in determining when, where, and how to achieve this during gestation in order to produce a healthy, successful pregnancy.

## Conflict of Interest Statement

Our work on IL-4 and IL-10 was funded by American Heart Association Grant in Aid 4480033.
